# Loss of Extracellular Signal-Regulated Kinase 1/2 in the Retinal Pigment Epithelium Leads to RPE65 Decrease and Retinal Degeneration

**DOI:** 10.1128/MCB.00295-17

**Published:** 2017-11-28

**Authors:** Aswin Pyakurel, Delphine Balmer, Marc K. Saba-El-Leil, Caroline Kizilyaprak, Jean Daraspe, Bruno M. Humbel, Laure Voisin, Yun Z. Le, Johannes von Lintig, Sylvain Meloche, Raphaël Roduit

**Affiliations:** a Department of Ophthalmology, University of Lausanne, Jules-Gonin Eye Hospital, Fondation Asile des Aveugles, Lausanne, Switzerland; b IRO, Institute for Research in Ophthalmology, Sion, Switzerland; c Department of Medicine Endocrinology and Harold Hamm Diabetes Center, University of Oklahoma Health Sciences Center, Oklahoma City, Oklahoma, USA; d Institute for Research in Immunology and Cancer, Department of Pharmacology and Program of Molecular Biology, Université de Montréal, Montreal, Quebec, Canada; e Electron Microscopy Facility, University of Lausanne, Lausanne, Switzerland; f Department of Pharmacology, School of Medicine, Case Western Reserve University, Cleveland, Ohio, USA

**Keywords:** AP-1, ERK1/2, photoreceptors, RPE65, retinal degeneration, retinoid, electron microscopy

## Abstract

Recent work suggested that the activity of extracellular signal-regulated kinase 1/2 (ERK1/2) is increased in the retinal pigment epithelium (RPE) of age-related macular degeneration (ARMD) patients and therefore could be an attractive therapeutic target. Notably, ERK1/2 pathway inhibitors are used in cancer therapy, with severe and noncharacterized ocular side effects. To decipher the role of ERK1/2 in RPE cells, we conditionally disrupted the *Erk1* and *Erk2* genes in mouse RPE. The loss of ERK1/2 activity resulted in a significant decrease in the level of RPE65 expression, a decrease in ocular retinoid levels concomitant with low visual function, and a rapid disorganization of RPE cells, ultimately leading to retinal degeneration. Our results identify the ERK1/2 pathway as a direct regulator of the visual cycle and a critical component of the viability of RPE and photoreceptor cells. Moreover, our results caution about the need for a very fine adjustment of kinase inhibition in cancer or ARMD treatment in order to avoid ocular side effects.

## INTRODUCTION

Age-related macular degeneration (ARMD) is the most common cause of blindness in individuals over 50 years of age. This pathology is characterized by the presence of soft and/or hard drusen (extracellular debris and deposits), hyper- and hypopigmentation of retinal pigment epithelium (RPE) cells, RPE and photoreceptor (PR) apoptosis, as well as choroidal neovascularization (CNV) (1). Two subgroups of ARMD can be distinguished: the dry form (geographic atrophy [GA]) and the wet form (exudative). The dry form is characterized by the formation of drusen between RPE cells and Bruch's membrane (2). This accumulation is toxic for RPE cells, altering some of their important functions. The wet form is linked to CNV affecting the subretinal macular region, which eventually results in a loss of central vision.

An accumulation of intracellular lipofuscin occurs in both forms of ARMD. One of the major components of lipofuscin is the retinoid derivative *N*-retinyl-*N*-retinylidene ethanolamine (A2E). A2E has a broad light absorption spectrum, with a peak in the blue range (∼430 nm). Therefore, it is a potent photoinducible generator of reactive oxygen species (ROS), which can damage proteins, lipids, and DNA of RPE cells (3–5). Even if new imaging techniques did not reveal any correlation between the distribution of A2E and lipofuscin fluorescence in the human RPE (6), it remains unclear whether metabolites or modified forms of A2E could be deleterious for the retina and RPE, especially because A2E accumulation is linked to an increase of lipofuscin levels with age (7). Moreover, the accumulation of A2E in phagolysosomes can lead to the inhibition of the turnover of endogenous proteins in cultured RPE cells by abolishing the pH gradient required for the normal function of these organelles (8). This damage leads to apoptosis and impaired RPE cell functions. In addition, previous work from our laboratory showed that A2E induces a strong decrease in extracellular signal-regulated kinase 1/2 (ERK1/2) activity in polarized ARPE19 and isolated mouse RPE cells, and the inhibition of ERK1/2 leads to a significant decrease in the level of retinal pigment epithelium-specific protein of 65 kDa (RPE65) (9).

Mitogen-activated protein kinases (MAPKs) are evolutionarily conserved protein kinases that transduce signals to regulate gene expression during cell proliferation, survival, and differentiation (10–12). There are two different groups of MAPKs: the conventional (ERK1/2/5, p38 kinases, and Jun N-terminal kinase 1/2/3 [JNK1/2/3]) and the atypical (ERK3/4, ERK7, and Nemo-like kinase [NLK]) MAPKs (13). Due to their important roles in cellular homeostasis, the abnormal regulation of conventional MAPK pathways has been linked to a wide range of diseases (12, 14–17). The ERK1/2 pathway in particular is commonly deregulated in human cancer, which has led to the development and clinical evaluation of several small-molecule inhibitors targeting components of this pathway (18, 19). Notably, the clinical benefit of these molecules is limited by mechanism-based side effects of blurred vision and altered light perception (20, 21).

Growing evidence indicates an important role of ERK1/2 signaling in retinal function. Retina maturation is associated with the activation of ERK1/2, which was proposed to play a survival role during development (22). We recently showed that ERK1/2 activity is upregulated in RPE65 knockout (RPE65-KO) mice (23) and is decreased when ARPE19 cells are exposed to UV stress (24). Regeneration of chicken embryo retinas was found to be associated with the fibroblast growth factor (FGF)/FGF receptor (FGFR)/MEK/ERK-dependent upregulation of the paired homeobox transcription factor PAX6 in the RPE (25). Interestingly, a recent clinical study by the Dridi et al. revealed increased ERK1/2 activity in the RPE of patients suffering from GA as well as in a mouse model of RPE degeneration induced by DICER1 depletion (26). These findings suggest a key role for ERK1/2 in ARMD and support the concept of ERK1/2 inhibition as a possible treatment for this disease (26). However, the ERK1/2 signaling pathway is complex, with multiple roles in differentiation, proliferation, and cell death pathways, depending on the cellular context. This complexity has been described in many studies (27, 28) and is a central question in order to understand the role of these kinases before considering whether to modulate their activity for therapeutic purposes.

Inhibitors of the ERK1/2 pathway used in cancer therapy may provoke ocular secondary effects (21), yet this treatment has been proposed for ARMD (26). In fact, little is known about the real impact of blocking these kinases in the mouse retina. Therefore, in order to obtain valuable insights about ERK1/2 inhibition and to understand the role of ERK1/2 signaling in the maintenance and survival of RPE cells, we established a mouse line with an RPE-specific knockout of the *Erk1* and *Erk2* genes (RPE double knockout [RPE-DKO]). Fundus analyses of mice with an RPE-specific loss of ERK1/2 showed macular depigmentation. Electroretinogram (ERG) analyses combined with retinoid measurements revealed dysfunctional vision as well as a significant decrease in the ocular retinoid content. Optical coherence tomography (OCT) analyses confirmed the disorganization of the retinal structure, and immunohistochemical analyses demonstrated RPE morphology alterations and the consequent loss of PRs. At the onset of retinal degeneration, the loss of ERK1/2 led to a specific decrease in the level of RPE65 with the mislocalization of lecithin retinol acyltransferase (LRAT). The diminution of RPE65 expression depended on the presence of an AP-1 site in the promoter region, as C-FOS and FRA-1 (Fos-related antigen 1) protein expression levels are decreased and binding to this AP-1 site is reduced in RPE-DKO mice.

## RESULTS

### RPE-specific loss of ERK1/2 causes vision impairment due to a deficit of retinoids.

In order to establish a mouse line with an RPE-specific knockout of the *Erk1* and *Erk2* genes, we used *Erk1^−/−^* mice with a conditional allele of *Erk2* (*Erk1^−/−^*; *Erk2^f/f^*) (29) crossed with transgenic mice carrying the human vitelliform macular dystrophy 2 (VMD2) promoter-directed reverse tetracycline-dependent transactivator (rtTA) and tetracycline-responsive element (TRE)-directed Cre (VMD2-rtTA/TRE-Cre) (30), called RPE-Cre mice, to obtain *VMD2-rtTA/TRE-Cre*; *Erk1^−/−^*; *Erk2^f/f^* mice. Doxycycline treatment of these mice leads to the disruption of *Erk2* specifically in RPE cells. The *VMD2-rtTA/TRE-Cre*; *Erk1^−/−^*; *Erk2*^Δ/Δ^ (RPE-DKO) line was then compared to the *Erk1^−/−^*; *Erk2^f/f^* line as control (CTL) mice.

**FIG 1:**
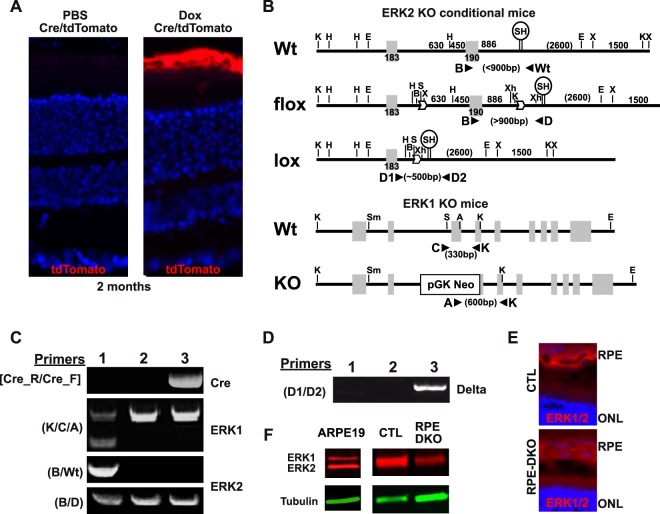
Generation and characterization of CTL and RPE-DKO mice. (A) tdTomato fluorescence of cryostat sections of fixed whole-mount eyes from Cre/tdTomato mice injected with either PBS or doxycycline (Dox). (B) Constructs used to generate Erk1-KO and Erk2 conditional mice (Erk1^−/−^ Erk2^f/f^) and primers used to genotype the mice. Wt, wild type. (C) Representative genotyping of Erk1^+/−^; Erk2^+/f^ mice (lane 1); Erk1^−/−^; Erk2^f/f^ mice, called CTL mice (lane 2); and VMD2-rtTA/TRE-Cre; Erk1^−/−^; Erk2^Δ/Δ^ mice, called RPE-DKO mice, when injected with Dox (lane 3). (D) The specific loss of Erk2 in the RPE is confirmed by the delta fragment present in genomic DNA isolated from RPE cells from Erk1^+/−^; Erk2^+/f^ mice (lane 1), Erk1^−/−^; Erk2^f/f^ mice, called CTL mice (lane 2); and VMD2-rtTA/TRE-Cre; Erk1^−/−^; Erk2^Δ/Δ^ mice, called RPE-DKO mice, when injected with Dox (lane 3). (E) Cryostat sections of fixed whole-mount eyes from CTL and RPE-DKO mice at 1 month, immunostained as indicated. (F) Western blot analysis of ERK1/2 expression in RPE protein lysates from CTL and RPE-DKO mice at 1 month. The ARPE19 protein lysate is used as a positive control for ERK1 expression.

**FIG 2:**
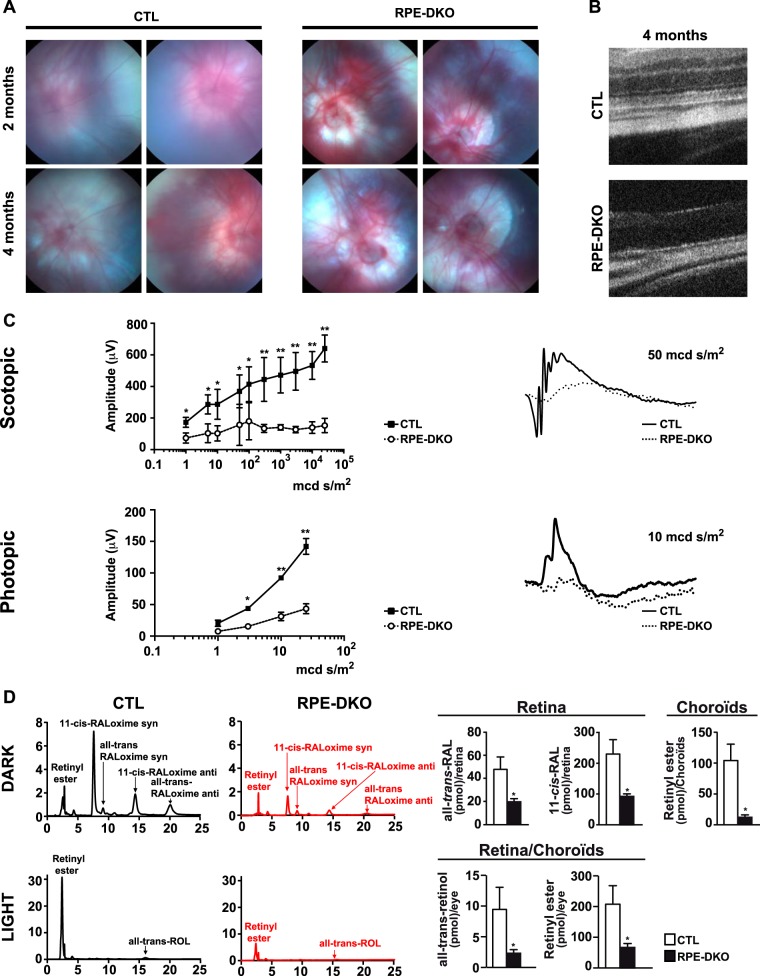
Loss of ERK1/2 in the RPE leads to vision impairment. (A) Representative fundus images of CTL and RPE-DKO mice at 2 or 4 months. (B) Representative OCT images of CTL and RPE-DKO mice at 4 months. (C) Graphs of the scotopic (*n* = 10) and photopic (*n* = 4) ERG responses (*b* wave) of CTL and RPE-DKO mice at 2 months. (*, *P* < 0.05; **, *P* < 0.001). The right panel shows the scotopic (50 mcd s/m^2^) and photopic (10 mcd s/m^2^) ERG responses of CTL and RPE-DKO mice. (D) Chromatographs and quantification of retinoids (retinol [ROL] and retinal [RAL]) measured in the retina and in the RPE-choroid from mice at 1 month, which were dark adapted for 16 h, or measured in whole eyes (containing the retina, RPE, and choroid) from mice (same conditions) exposed to 1,000 mA of light for 2 h.

We first analyzed the visual function and retinal structure of RPE-DKO mice in comparison to CTL mice. The doxycycline (Dox)-induced expression of Cre in RPE cells was verified by crossing RPE-Cre animals with tdTomato mice (31) to generate RPE-Cre-tdTomato mice. Two intraperitoneal (i.p.) injections of 10 μg Dox, 1 week apart, at 2 months of age triggered the consistent expression of Cre specifically in RPE cells (Fig. 1A). This protocol was followed to induce Cre protein expression in 2-month-old RPE-DKO mice, while 2-month-old CTL mice were injected with phosphate-buffered saline (PBS). The genotypes of all mice were confirmed by PCR amplification of *Cre*, *Erk1* KO, and *Erk2* floxed alleles (Fig. 1B and C); the deletion of *Erk2* in the RPE was confirmed by an analysis of RPE genomic DNA, as evidenced by the presence of the delta (Δ) band (Fig. 1D). Immunostaining showed a decreased ERK2 level in the RPE of RPE-DKO mice at 1 month in comparison to the level in control mice (Fig. 1E). In addition, Western blot analysis of RPE protein lysates clearly showed the absence of ERK1 in both RPE-DKO and CTL mice and a decreased ERK2 level only in RPE-DKO mice (Fig. 1F). Fundus analysis showed an apparent depigmentation of RPE-DKO eyes (Fig. 2A). OCT analyses performed on RPE-DKO mice at 4 months revealed the degeneration of the retina with a significant reduction of all retinal and choroidal layers (Fig. 2B). In accordance with this result, ERG analyses revealed a significant impairment of vision in RPE-DKO mice at 2 months, as revealed by the lower amplitude of the *b* wave in both the scotopic and photopic stimulations (Fig. 2C). Scotopic patterns (50 mcd s/m^2^) demonstrated the absence or severe reduction of *a* and *b* waves in RPE-DKO mice in comparison to CTL mice, while photopic patterns (10 mcd s/m^2^) showed an absence of stimulation in RPE-DKO mice (Fig. 2C, right). This clearly indicates impairments in both the cone and rod photoreceptor responses to light stimuli in RPE-DKO animals.

The severe vision damage and retinal degeneration observed in RPE-DKO mice led us to measure the relative amounts of visual cycle intermediates. Mice that had been dark adapted for 12 h were enucleated under dim light. The retina was separated from the choroid and subjected to high-performance liquid chromatography (HPLC) analyses as previously described (32). The loss of ERK1/2 in the RPE led to a significant decrease in the level of ocular retinoids. Levels of retinyl esters in the choroid (dissected to include RPE cells) were significantly reduced. In the retinas of dark-adapted eyes, decreases of more than half of the amounts of 11-*cis*-retinal and all-*trans*-retinal were observed (Fig. 2D). We further intended to measure how light influences the ocular retinoid composition of these mice. Thus, we exposed the mice to bright light, where the turnover of the visual cycle is enhanced. As expected, we observed increases in the amounts of all-*trans*-retinol and retinyl ester(s) after light treatment. Notably, the levels of both retinoids were significantly lower in RPE-DKO mice than in controls, indicating an impairment of the visual cycle in both dark-adapted and light-exposed mice (Fig. 2D). We also noted, by immunostaining analysis, a decrease in the expression level of the stimulated by retinoic acid 6 (STRA6) protein, the membrane receptor for retinol binding protein found in the RPE in RPE-DKO mice at 1 month (data not shown). This decrease suggests that a reduction of retinoid uptake from the circulation could contribute to the diminished ocular retinoid levels in RPE-DKO mice.

### RPE-specific loss of ERK1/2 causes significant thinning of the ONL/INL.

**FIG 3:**
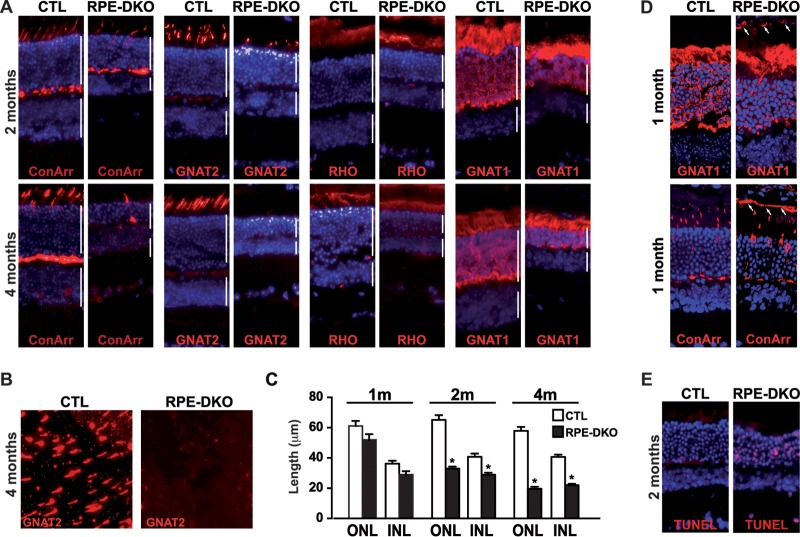
Loss of ERK1/2 in the RPE leads to photoreceptor degeneration. (A) Cryostat sections of fixed whole-mount eyes from CTL and RPE-DKO mice at 2 or 4 months, immunostained against different rod and cone markers, as indicated. (B) Flat-mount retinal preparation from CTL and RPE-DKO mice at 4 months, immunostained as indicated. (C) Outer nuclear layer (ONL) and inner nuclear layer (INL) lengths measured manually by using ImageJ. Data represent means ± standard errors of the means of results from four independent experiments. *, *P* < 0.05. (D) GNAT1 and cone arrestin stainings from CTL and RPE-DKO mice at 1 month. The presence of both photoreceptor markers was observed in the RPE of RPE-DKO mice (white arrows). (E) TUNEL staining of cryostat sections of fixed whole-mount eyes from CTL and RPE-DKO mice at 2 months.

**FIG 4:**
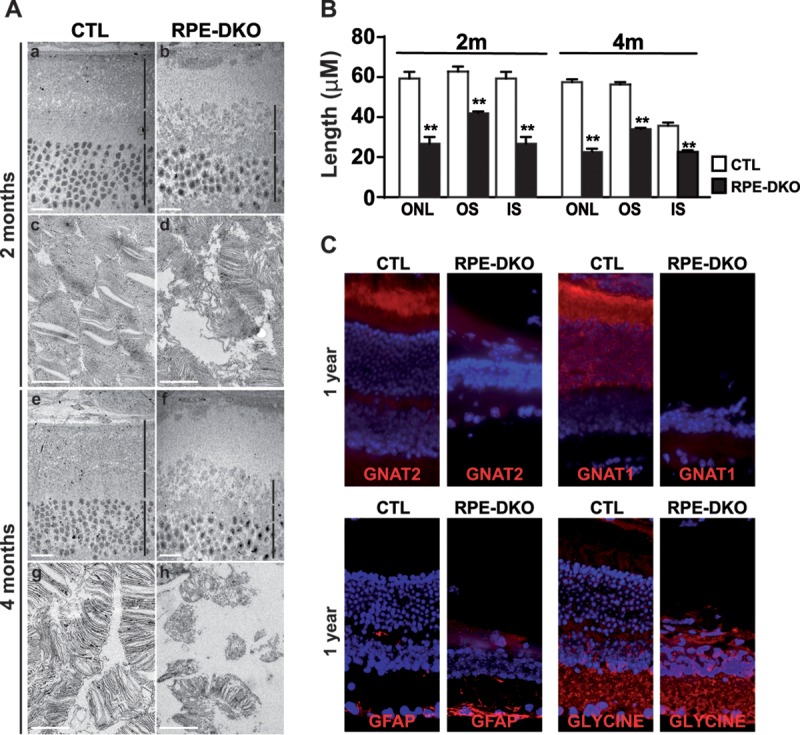
Decreases of outer nuclear layer (ONL), outer segment (OS), and inner segment (IS) lengths from the start of degeneration to complete absence in RPE-DKO mice at 1 year. (A) Electron microscopy images of retina-RPE layers from CTL and RPE-DKO mice at 2 or 4 months. The bottom panel is a magnification of the OS layer of photoreceptors (PR). (B) Measurement of ONL, OS, and IS from electron microscopy images. Data represent means ± standard errors of the means of results from 3 experiments. **, *P* < 0.008. (C) Cryostat sections of fixed whole-mount eyes from CTL and RPE-DKO mice at 1 year, immunostained as indicated.

Visual impairment along with retinal dysfunction led us to examine the photoreceptor structure in RPE-DKO mice. Staining of various cone and rod markers was performed to analyze the integrity of the distinct retinal layers. Retinal degeneration was observed only in RPE-DKO mice. As controls, we used ERK1^−/−^; Erk2^f/f^ mice treated with PBS (CTL), ERK1^−/−^; Erk2^f/f^ mice treated with Dox, and VMD2-rtTA/TRE-Cre; ERK1^−/−^; Erk2^f/f^ mice treated with PBS; none of these mice showed retinal degeneration (data not shown). No toxic effect of Dox was observed in CTL (data not shown) or in Cre-tdTomato (Fig. 1A) mice. RPE-DKO mice exhibited reductions in the levels of cone markers as well as in the outer nuclear layer (ONL) and the inner nuclear layer (INL) thicknesses. Levels of cone arrestin and GNAT2 showed marked decreases in RPE-DKO mice at 2 months, and this effect was more pronounced at 4 months (Fig. 3A). The important loss of cone markers occurred before the disappearance of the respective rod markers, as shown by immunostaining of GNAT2 (Fig. 3A and B). The levels of the rod markers rhodopsin and GNAT1 were slightly decreased in RPE-DKO mice, principally in the ONL. In addition, the outer segment (OS) length was already smaller in RPE-DKO mice at 2 months (Fig. 3A). Retinal degeneration could explain the absence of a marked reduction of the GNAT1 protein expression level shown by Western blot analysis, because both GNAT1 and tubulin levels were decreased, so the ratio was not changed. Measurement of the thicknesses of the ONL and INL confirmed clear decreases in both cases, underlining the devastating effect of the loss of ERK1**/**2 on RPE cells (Fig. 3C). In fact, ONL and INL thicknesses were reduced by 50% after only 2 months of ERK1**/**2 loss and by up to 70% after 4 months (Fig. 3C). We found staining of both GNAT1 and CONE-ARRESTIN in the RPE of RPE-DKO mice at 1 month, while no staining was observed in the RPE of CTL mice, as expected (Fig. 3D); this information supports the hypothesis of a defect in the phagocytosis process in these mice. Interestingly, ONL and INL degenerations correlated with the level of cell death, as demonstrated by terminal deoxynucleotidyltransferase-mediated dUTP-biotin nick end labeling (TUNEL) assays performed on RPE-DKO and CTL mice (Fig. 3E). The decreases of the ONL and OS thicknesses as well as the structural disorganization of the entire retina were confirmed by electron microscopy (EM) imaging, revealing the disruption of the inner segment (IS) and OS of photoreceptor cells (Fig. 4A). This disruption and loss of the OS in particular were quantified by measuring the ONL, OS, and IS lengths (Fig. 4B). A follow-up study of the mice for 1 year showed the disappearance of the ONL and a drastic thinning of the INL, with no staining for cones or rods. The only persistent staining was for glial fibrillary acidic protein (GFAP) in the ganglion cell layer (GCL) and glycine in the inner plexiform layer (IPL) (Fig. 4C).

### RPE-specific loss of ERK1/2 causes specific reductions in levels of cone markers.

**FIG 5:**
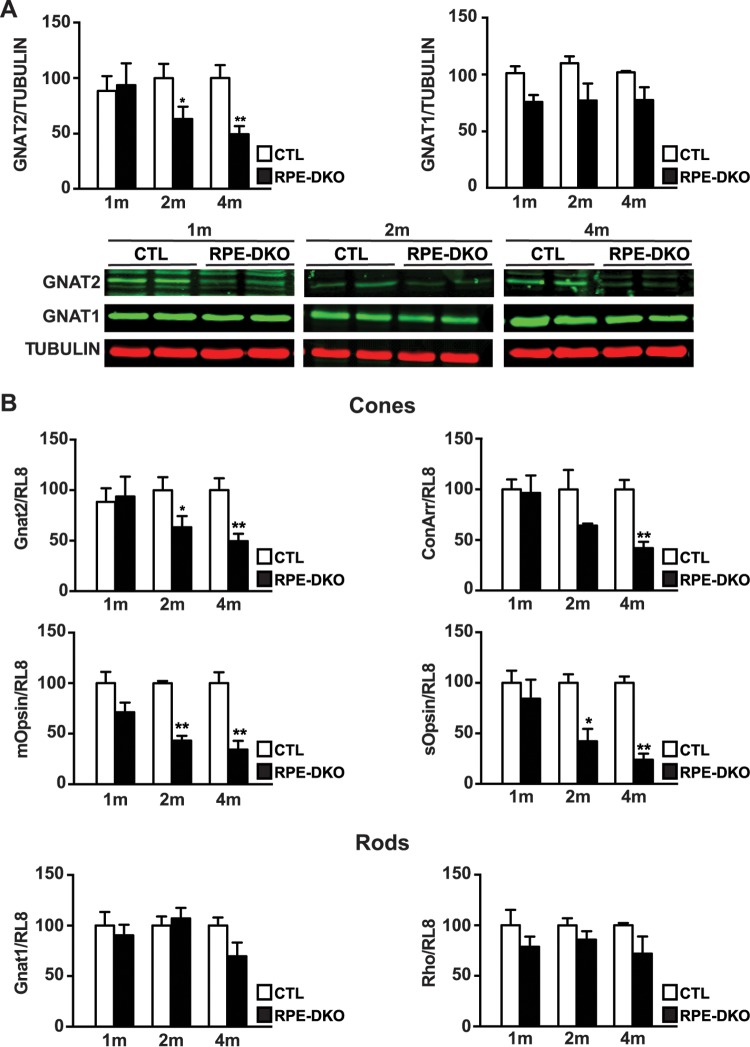
Loss of ERK1/2 in the RPE causes specific reductions in the levels of cone markers. (A) Representative images and quantification graphs of retinal protein lysates from CTL and RPE-DKO mice at 1, 2, or 4 months. Data represent means ± standard errors of the means of results from 5 independent experiments (*, *P* < 0.02; **, *P* < 0.001). (B) Quantification graphs for qPCR performed on retinal extracts from CTL and RPE-DKO mice at 1, 2, or 4 months. RL8 was used as an internal control to normalize RNA expression. Results are expressed as a percentage of the value for CTL mice and as means ± standard errors of the means of results from 4 independent experiments (*, *P* < 0.05; **, *P* < 0.003).

Additional biochemical analyses were carried out in order to verify the loss of cone photoreceptors. Western blot analysis of retinas from CTL and RPE-DKO mice showed that the level of GNAT2, a specific cone marker, was significantly reduced at 2 months and was reduced by more than 60% at 4 months (Fig. 5A). However, at 4 months, we observed a marked decrease in both ONL and INL thicknesses, which could not be due only to cone photoreceptor loss. Indeed, at 2 months, the level of GNAT1 was already slightly affected, while at 4 months, the difference was visible even if not significant. At 4 months, the decreases in the levels of photoreceptor proteins were mirrored by reductions in their mRNA levels. Quantitative PCR (qPCR) showed that the levels of specific cone markers, including *Gnat2*, *ConeArrestin*, *mOpsin*, and *sOpsin*, were significantly decreased at 2 months and were decreased further by 60 to 80% at 4 months (Fig. 5B). In agreement with data from Western blot studies, the levels of the specific rod markers *Gnat1* and *Rho* showed no difference at 2 months but declined at 4 months, although the reduction was not statistically significant. Retinal degeneration could explain the absence of a significant reduction in the level of GNAT1 protein expression shown by Western blotting, because both GNAT1 and TUBULIN levels were decreased, so the ratio was not changed.

### Loss of ERK1/2 in the RPE causes RPE ultrastructural damage.

**FIG 6:**
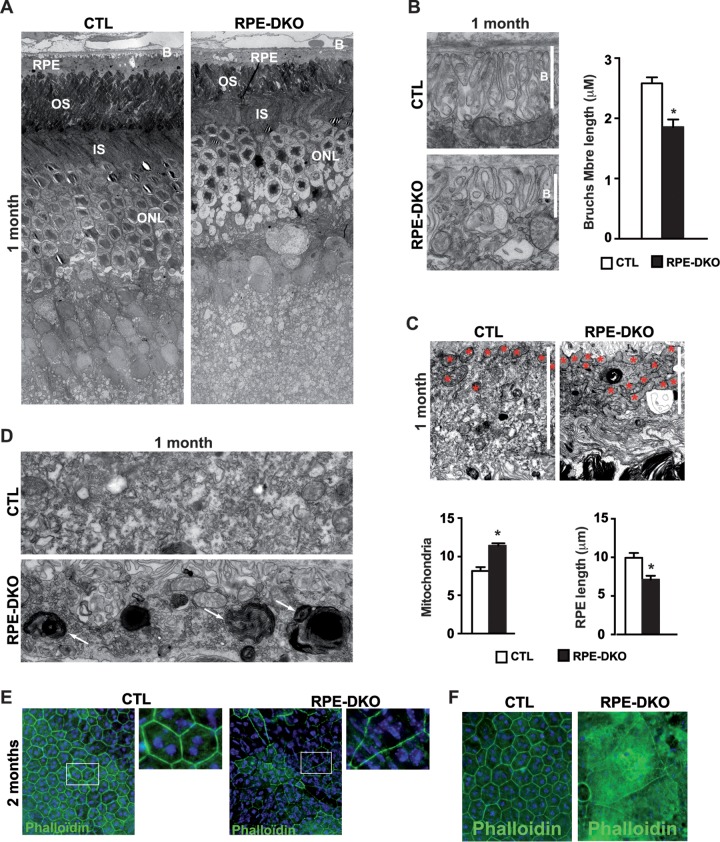
Loss of ERK1/2 in the RPE leads to massive ultrastructural changes in RPE-DKO mice at 1 month. (A) Representative electron microscopy images showing different retinal and RPE cell layers (B, Bruch's membrane; RPE, retinal pigment epithelium; OS, outer segment; IS, inner segment; ONL, outer nuclear layer). (B) Representative electron microscopy image of Bruch's membrane (Mbre) and the underlying RPE cells and measurement of the length of Bruch's membrane (*, *P* < 0.0001). (C) Representative electron microscopy images of the RPE cell layer and the underlying OS of the PR, measurement of the RPE length, and quantification of the area of mitochondria (stars, mitochondria) (*, *P* < 0.015). (D) Electron microscopy images showing the accumulation of membrane-enriched phagolysosomes in the RPE of RPE-DKO mice in comparison to CTL mice (white arrows). (E and F) RPE flat mounts from CTL and RPE-DKO mice at 2 months, immunostained against phalloidin and counterstained with DAPI.

The loss of vision as well as the pronounced retinal degeneration in these mice prompted us to examine the state of the RPE ultrastructure in RPE-DKO mice at 1 month by EM imaging. An overview of the RPE-retina cell layers showed massive shrinkage of the RPE cell layer, a disruption of Bruch's membrane (BM), and PR degeneration (Fig. 6A). In order to characterize the damage to the RPE-retinal layer in more detail, different layers were observed separately and quantified. The BM ultrastructure was clearly disrupted, and the thickness of the membrane was significantly reduced (Fig. 6B). The RPE cell layer exhibited massive shrinkage, as evidenced by the significant reduction of its length (Fig. 6C). The ultrastructural damage to the RPE cell layer was accompanied by the accumulation of mitochondria at the basolateral layer membrane, as demonstrated by the significant increase of the organelle area when normalized to the area of the cytosol (Fig. 6C). The RPE cell layer of RPE-DKO mice contained structures enriched in membranes that resembled phagolysosomes of the OS of the PR (Fig. 6D). This indicates that phagocytosis of OS, one of the major functions of RPE cells, is impaired in RPE-DKO mice. Confirmation was obtained by the presence of GNAT1 and CONE-ARRESTIN in the RPE of RPE-DKO mice at 1 month (Fig. 3D). Ultrastructural analysis demonstrated that the RPE-specific loss of ERK1/2 caused massive alterations to the ultrastructures of not only the RPE cell layer but also the BM and the retinal cell layer. The ultrastructural RPE damage in RPE-DKO mice was confirmed by flat-mount RPE-choroid analysis. Phalloidin staining revealed a disorganized RPE structure along with an increased RPE cell size, including multinucleated cells (Fig. 6E). A total loss of RPE cells was observed in parts of the retinas of RPE-DKO mice at 2 months (Fig. 6F).

### ERK1/2 directly regulates RPE65 levels.

**FIG 7:**
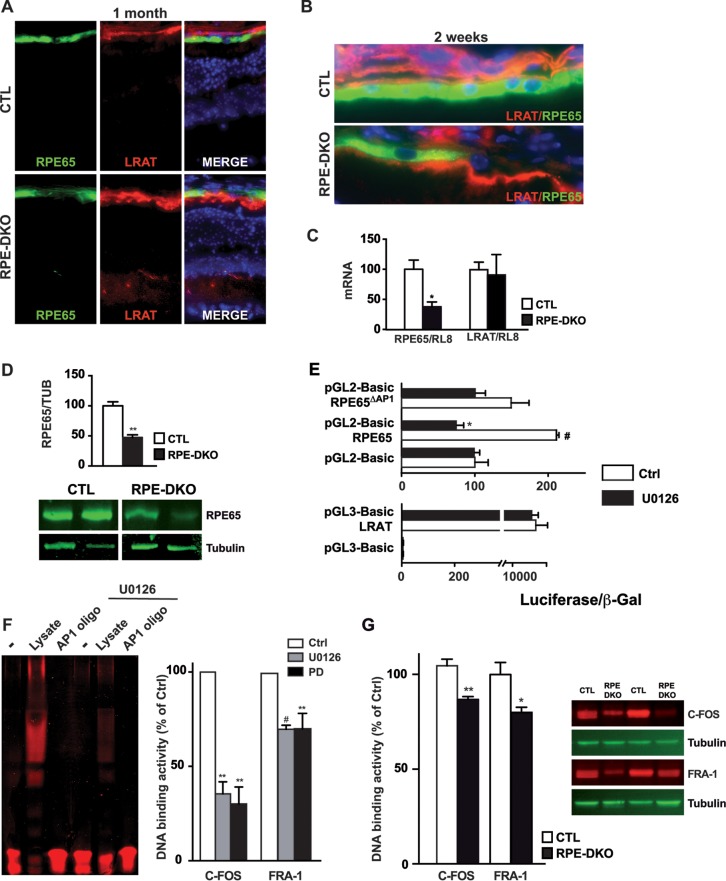
ERK1/2 directly regulates RPE65 expression. (A) Cryostat section of fixed whole-mount eyes from CTL and RPE-DKO mice at 1 month, immunostained as indicated. (B) Cryostat section of fixed whole-mount eyes from CTL and RPE-DKO mice at 2 weeks, immunostained as indicated. (C) qPCR analysis of mRNAs for the genes indicated, extracted from the RPE-choroids of CTL and RPE-DKO mice at 1 month. RL8 was used as an internal control to normalize RNA expression. Results are expressed as a percentage of the value for CTL mice and as means ± standard errors of the means of results from 3 retinas. *, *P* < 0.03. (D) Representative immunoblot and quantification of RPE protein lysates from CTL and RPE-DKO mice at 1 month, immunoblotted as indicated. **, *P* < 0.0002 (*n* = 5). (E) Luciferase assay for both RPE65 and LRAT promoters. HEK293 cells were transfected and treated as indicated. Luciferase fluorescence was normalized to β-Gal fluorescence. Data represent means ± standard errors of the means of results from 4 independent experiments. *, *P* < 0.05 versus pGL2-Basic-RPE65; #, *P* < 0.05 versus pGL2-Basic. (F) EMSA of the AP-1 complex in nuclei from ARPE19 cells treated or not with U0126. AP-1 DNA binding analysis of nuclear extracts of ARPE19 cells treated with either U0126 or PD0325901. Data represent means ± standard errors of the means of results from 3 independent experiments, expressed as a percentage of the value for the control. *, *P* < 0.02; **, *P* < 0.002. (G) AP-1 DNA binding analysis of whole-cell extracts from mice at 1 month. Data represent means ± standard errors of the means of results from 3 independent experiments, expressed as a percentage of the control. *, *P* < 0.015; **, *P* < 0.002 (*n* = 6). Also shown is a representative immunoblot of RPE protein lysates from CTL and RPE-DKO mice at 1 month, showing the expression of C-FOS and FRA-1; tubulin was used as a control.

We previously showed that the inhibition of the ERK1/2 pathway in ARPE19 cells as well as in isolated mouse RPE cells leads to a reduction of *Rpe65* mRNA expression (9). This decrease was confirmed by RPE65 immunostaining in RPE-DKO mice at 1 month and at 2 weeks, respectively (Fig. 7A and B). In addition, we observed a mislocalization of LRAT, the enzyme upstream of RPE65 in the visual cycle, to the apical side of RPE cells in RPE-DKO mice, instead of the basolateral side as observed in CTL mice (Fig. 7A and B). qPCR analysis confirmed the results of immunostaining and revealed that RPE65 but not LRAT expression was significantly decreased in RPE cells of RPE-DKO mice at 1 month (Fig. 7C). The RPE65 protein expression level, normalized to the TUBULIN expression level, was significantly reduced in RPE-DKO mice at 1 month (Fig. 7D). Notably, the promoter of *RPE65* has been shown to contain an AP-1 site, which is under the transcriptional control of the AP-1 family of proteins (33). We thus carried out an *in vitro* luciferase reporter assay using RPE65 (33) or LRAT (34) promoter constructs. Luciferase activity was detected with both promoters transfected into the HEK293 cell line (Fig. 7E). Treatment with the MEK1/2 inhibitor U0126 caused a significant decrease of the activity of the RPE65 promoter, while no change in activity was observed when the luciferase reporter was driven by the LRAT promoter. The addition of U0126 did not significantly affect luciferase activity when the AP-1 site in the *RPE65* promoter reporter construct was deleted, indicating that it is mandatory for regulation by ERK1/2. Next, we set out to identify the downstream effectors of ERK1/2 that regulate this AP-1 site. Nuclear proteins of ARPE19 cells showed strong binding to the AP-1 sequence, which was reduced in the presence of a competitor AP-1 oligonucleotide as well as by treatment with U0126 (Fig. 7F). In order to identify which factors regulated by ERK1/2 are part of the AP-1 complex, nuclear extracts from ARPE19 cells were subjected to an AP-1 binding enzyme-linked immunosorbent assay (ELISA) that included individual AP-1 family transcription factors. The inhibition of the ERK1/2 pathway in ARPE19 cells, using either U0126 or PD0325901, led to decreased C-FOS and FRA-1 protein binding to the AP-1 sequence (Fig. 7F), whereas the inhibitors had no effect on c-JUN, JUN-B, and JUN-D binding (data not shown). In order to investigate whether this effect is maintained in RPE-DKO mice, whole-cell protein lysates from isolated RPE cells were subjected to an AP-1 DNA binding assay. Indeed, the binding of C-FOS and FRA-1 to AP-1 was significantly reduced (Fig. 7G), and C-FOS and FRA-1 protein expression levels were decreased in the RPE of RPE-DKO mice at 1 month (Fig. 7G). Taken together, data from analyses of cell cultures and the mouse model argue in favor of a direct regulation of *RPE65* gene expression by the ERK1/2 pathway via the binding of C-FOS and FRA-1 complexes to the AP-1 site in the upstream promoter region.

## DISCUSSION

In order to evaluate the potential use of ERK1/2 pathway inhibitors in ARMD treatment and to better understand the ocular side effects observed in cancer patients treated with such inhibitors, we disrupted ERK1/2 expression specifically in RPE cells of mice. We observed two major consequences. The first consequence is the downregulation of RPE65, the mislocalization of LRAT, and the depletion of the retinoid content. This already occurs 1 month after ERK1/2 depletion, when retinal degeneration is just beginning to be detected, as shown by chromatin compaction in ONL cells as well as the shortening of the photoreceptor OS. The second consequence includes changes in the morphology of the RPE and in the RPE cell death process, which appears later, 2 months after Cre induction, when most of the hallmarks of retinal degeneration are visible in the eyes of RPE-DKO mice.

RPE65 is an enzyme involved in vitamin A metabolism in RPE cells (35). This key protein of the visual cycle catalyzes the transformation of all-*trans*-retinyl to 11-*cis*-retinol. Mutations in RPE65 are associated with several retinal disorders, including retinitis pigmentosa (RP), Leber congenital amaurosis (LCA) (36), and early-onset retinal dystrophy (RD) in children (37). Much effort has been devoted to deciphering the roles of various genetic mutations that are linked to these diseases (38). However, little is known about how the activity of RPE65 is regulated at the RPE cell level. Several studies analyzed the promoter region of the *RPE65* gene (33, 39), but little is known about the factors involved in its transcriptional regulation. Retinoic acid has been suggested to participate in the downregulation of *RPE65* (40) and of fatty acid transporter protein 4 (FATP4) and elongation of very-long-chain fatty acid protein 1 (ELOVL1) (41). Recently, Masuda and colleagues provided evidence for an involvement of sex-determining region Y box-containing gene 9 (SOX9) in the regulation of the transcription of visual cycle genes, including RPE65, retinaldehyde binding protein 1 (RBP-1) and retinal G-protein-coupled receptor (RGR). They showed that SOX9 acts synergistically with orthodenticle homeobox 2 (OTX2) to activate *RPE65* gene expression (42). Here we identify another critical upstream regulatory factor for *RPE65*. We demonstrate that the disruption of ERK1/2 specifically in RPE cells leads to a marked decrease of *RPE65* expression that occurs through an AP-1 site present in the promoter region of the *RPE65* gene. The inhibitory effect of U0126 on reporter gene expression was completely abrogated when the AP-1 site was removed, even if we cannot exclude that other regulatory elements close to the AP-1 sequence were also deleted from the construct; this could explain the low promoter activity observed in the absence of the AP-1 sequence. The key roles of C-FOS and FRA-1 were confirmed by Western blot analysis and by an AP-1 DNA binding assay. Interestingly, ERK1/2 and SOX9 signaling pathways were recently shown to interact in urothelial carcinoma (43) and zebrafish sex determination (44). Moreover, the activation of ERK1/2 is associated with the activation of the Wnt/β-catenin pathway (45, 46), which plays a key role in the expression of the RPE-specific transcription factors microphthalmia-associated transcription factor (MITF) and OTX2 (47). In conjunction with protein paired box 6 (PAX6), MITF and OTX2 are key factors in RPE development (48).

ERG analysis combined with the measurement of ocular retinoid levels revealed the impaired vision of RPE-DKO mice. The significant decrease in the level of retinoids and histological evidence of retinal degeneration explain the absence of an ERG response in these mice. The RPE plays critical roles in providing nutrients to the adjacent retina and in the recycling of the visual chromophore. Upon absorption of light, 11-*cis*-retinal isomerizes to all-*trans*-retinal in the outer segments of the photoreceptors, and the RPE is necessary for the subsequent regeneration of the chromophore throughout the visual cycle (49). The loss of ERK1/2 leads to a decrease in the level of RPE65, and therefore, one would expect retinyl ester(s) to accumulate in RPE cells, as reported previously for RPE65 mutant mice (50). However, for RPE-DKO mice, we observed a significant decrease in the level of retinyl ester(s) in both dark- and light-exposed eyes. Therefore, in addition to RPE65 downregulation, which could explain the decrease in the level of 11-*cis*-retinal, other pathological alterations in the RPE must account for the decreased levels of ocular retinoids. We found a mislocalization of LRAT, which is expressed mostly at the apical region of RPE cells in RPE-DKO mice, in comparison to its normal basolateral localization (51). This mislocalization could affect LRAT activity and explain the decreased levels of all-*trans*-retinyl esters. Moreover, we also measured a lower expression level of STRA6, an RPE membrane receptor for the retinol binding protein responsible for retinyl ester transport. Because STRA6 and LRAT work together to retrieve retinoids from the blood circulation (52), such diminution may explain the reduction of the retinoid content observed in RPE-DKO mice. These results phenocopy some of the phenotypes observed for Stra6^−/−^ mice, including low retinoid content, the lack of an ERG response, decreases of BM and RPE lengths, and altered morphology of RPE cells (53).

The alteration of the RPE morphology in RPE-DKO mice was already visible at 1 month. Whole mounts of RPE from CTL mice show mono- and binucleated hexagonal RPE cells with an apex shared by three cells, while RPE-DKO mice exhibit an unusual RPE morphology, with a total disappearance of the RPE in some parts of the retina 2 months after ERK1/2 depletion, whereas other parts display large cells with an irregular shape and multinucleation (Fig. 6). RPE multinucleation, which has been reported for humans (54), could result from a cytokinesis defect. The involvement of cytokinesis defects as well as RPE dysfunctions and RPE cell death in aging eyes was recently described (55). As the ERK1/2 MAPK pathway plays a key role in cell proliferation, we can envisage that these kinases might be involved in the process. The sustained activation of ERK1/2 is necessary for the progression from G_1_ phase to S phase and is associated with the upregulation of proliferation-associated genes and the downregulation of antiproliferative genes (56). Moreover, ERK1/2 signaling is necessary to allow the entry of RPE cells into cell cycling and RPE cell proliferation (57). In addition to its role in cell cycle control, ERK1/2 is also implicated in centrosome orientation (58), which is crucial for cytokinesis. Thus, we postulate that a disruption of ERK1/2 in the RPE induces defects in cell cycle progression, leading first to the multinucleation of the RPE and then to RPE cell death. Whereas central RPE cells are senescent, peripheral cells proliferate, and ERK1/2 could be important for their maintenance (59, 60). Retinal regeneration in the chicken embryo was shown to be dependent on the FGF/FGFR/MEK/ERK-dependent upregulation of PAX6 (25). The activation of the ERK1/2 pathway was also responsible for the 15-deoxy-Δ^12,14^-prostaglandin J_2_ (dPGJ_2_)-dependent protection of RPE cells from oxidative injury (61). These data point not only to a protective role of the ERK1/2 pathway in RPE cells but also to its participation in the maintenance and proliferation of these cells, at least in the peripheral region. Our data indicate that the ablation of ERK1/2 in 2-month-old adult RPE cells almost completely abrogated vision by initiating early cone-specific degeneration, followed by rod impairment. Surprisingly, we observed a decrease of GNAT1 immunostaining in Cre-DKO mice at 2 and 4 months, which was not observed by either qPCR or Western blot analysis. At the same time, we clearly detected a decrease in the ONL length, and thus, even if the total GNAT1 retinal content decreases, the tubulin abundance decreases in parallel so that the GNAT1/tubulin ratio is not modified. One outstanding question is why cones are more affected than rods in this model. The dependence of cone PR survival on RPE65 was reported previously (62); therefore, the direct regulation of RPE65 by ERK1/2 could account, at least in part, for the severe cone dystrophy found in RPE-DKO mice. Moreover, inadequate 11-*cis*-retinal production has been shown to be associated with cone degeneration, although the mechanisms are currently not well understood (63–65). Measurement of retinoid levels specifically in the retina of RPE-DKO mice revealed a significant decrease in the level of 11-*cis*-retinal, which could contribute to rapid cone degeneration. This finding suggests that the cone-specific effect seen at 2 months could be directly attributed to the role of ERK1/2 in regulating RPE65 activity and 11-*cis*-retinal production that are critical for cone PR survival.

Previously reported observations demonstrated that PR degeneration in Rpe65^−/−^ mice caused a massive activation of ERK1/2 in the GCL of the retina (23), probably in order to protect the retina against stress induced by the absence of RPE65. However, we recently showed that in polarized ARPE19 cells and isolated mouse RPE, A2E treatment decreases phospho-ERK1/2 significantly, along with a reduction of the RPE65 level. In addition, the inhibition of the ERK1/2 pathway by U0126 also induces a significant decrease in the level of *RPE65* expression (9). The role of ERK1/2 in RPE cells has not been the subject of detailed investigation. Our study showing that the loss of ERK1/2 leads to RPE cell death, retinal atrophy, and degeneration strongly supports a significant role for ERK1/2 in the maintenance and survival of RPE cells. Defects of RPE cells and RPE cell death will lead to PR degeneration. Consistently, these features were observed in a mouse model of genetic RPE ablation using mice that expressed inducible diphtheria toxin A (DTA) specifically in the RPE. In this RPE^CreER^/DTA model, functional analysis showed very low scotopic and photopic ERG responses as well as PR degeneration (66). Accordingly, interactions of PRs with the RPE are essential for PR survival (67).

The very rapid INL degeneration observed in RPE-DKO mice is more difficult to explain. Even if Cre expression is observed specifically in RPE cells, as shown for Cre-tdTomato mice (Fig. 1) and as previously described (30), we also noted the ectopic expression of Cre in IPL vessels (but not in choroidal vessels) in certain mice. Therefore, we cannot totally exclude that ERK1/2 was also depleted in some IPL vessels, which may impact INL degeneration. We also hypothesize that the absence of key factors normally secreted by the RPE may influence INL degeneration. Further analyses are required in order to decipher the mechanisms leading to INL degeneration in ERK1/2-depleted mice.

Several MEK1/2 inhibitors have been clinically evaluated for cancer therapy, but some of these early-phase trials were stopped prematurely because of toxicity issues, including various ocular adverse effects (21, 68–70). Treatments last approximately 3 weeks and are repeated every month (71, 72). Such long-term treatment with multiple doses of highly potent MEK inhibitors lead to a marked and sustained inhibition of ERK1/2 activity, phenocopying in part the impact of the double knockout. We observed a significant decrease of the RPE65 expression level already at 2 weeks (Fig. 7B), while mRNA and protein levels of this RPE marker were decreased by about 50% at 1 month (Fig. 7C and D). Our study provides evidence for a key role of ERK1/2 signaling in the eye and, more specifically, in RPE cells. The ablation of ERK1/2 signaling reduces *RPE65* expression levels, leads to decreased retinoid levels, affects the RPE and the retinal structure, and induces retinal degeneration. Therefore, the use of ERK1/2 pathway inhibitors in ARMD treatment, as recently suggested (26), has to be reevaluated, taking into consideration the findings reported here. Fine-tuning of ERK1/2 inhibition will be necessary in order to block only the negative effects of high kinase activity and restore low ERK1/2 activity without impacting the role of this kinase in RPE cells.

## MATERIALS AND METHODS

### Animals.

Studies involving mice adhered to the Association for Research in Vision and Ophthalmology (ARVO) statement for the use of animals in ophthalmic and vision research (https://www.arvo.org/content-detail/level2-about2/policies2/statement-for-the-use-of-animals-in-ophthalmic-and-visual-research/) and were approved (permit number VD3023) by the Veterinary Service of the State of Vaud (Switzerland). Animals were maintained in a 12-h-light/12-h-dark cycle and had unlimited access to food and water. The generation of *Erk1^−/−^*/*Erk2^fl/fl^* mice was reported previously (29). The RPE-specific loss of ERK1/2 (*VMD2-rtTA/TRE-Cre*; *Erk1^−/−^*; *Erk2*^Δ/Δ^ [RPE-DKO]) was created by crossbreeding an *Erk1^−/−^*/*Erk2^f/f^* mouse with a mouse expressing the Cre recombinase driven by the human VMD2 (RPE-Cre) promoter and inducible by doxycycline (30) to first obtain *VMD2-rtTA/TRE-Cre*; *Erk1^−/−^*; *Erk2^f/f^* mice. Two months after birth, VMD2-rtTA/TRE-Cre; Erk1^−/−^; Erk2^f/f^ mice were then injected twice, 1 week apart, with 10 μg of Dox in 500 μl PBS in order to induce the Cre recombinase; we call these mice RPE-DKO mice. As a control, Erk1^−/−^/Erk2^f/f^ mice were injected in a similar way with PBS; we call these mice CTL mice. Every time period mentioned in the experiments refers to the amount of time after Dox injection (e.g., an RPE-DKO mouse at 2 months is a 4-month-old mouse treated after 2 months and analyzed 2 months later, after Cre induction and ERK1/2 depletion). Cre-tdTomato mice were created by crossbreeding VMD2-rtTA/TRE-Cre mice with tdTomato mice (31) to set up the Dox injection protocol and visualize Cre expression. Genotyping of mice was carried out by using PCR analysis with genomic DNA isolated from ear punches (Direct PCR [Ear]; Viagen). All the mice were verified to have the mutant Rd1-negative genotype. The mice were processed for fundus, OCT, and ERG analyses or sacrificed for other functional assays 2 weeks, 1 month, 2 month, 4 months, or 1 year after the second injection.

### Cell culture.

HEK293T cells were cultured in high-glucose Dulbecco's modified Eagle's medium (DMEM) containing 25 mM HEPES. The cells were passaged every 2 to 3 days. A total of 600,000 cells were seeded into a P60 plate and transfected after 24 h by using a calcium-phosphate transfection kit (GE Healthcare) with the indicated plasmids, according to the manufacturer's instructions. When indicated, cells were treated with 10 nM U0126 (Cell Signaling Technology) or 2 μM PD0325901 (Cell Signaling Technology) for 24 h and lysed with lysis buffer (20 mM morpholinepropanesulfonic acid [MOPS] [pH 7.0], 2 mM EGTA, 5 mM EDTA, 30 mM sodium fluoride, 60 mM β-glycerophosphate [pH 7.2], 20 mM sodium pyrophosphate, 1 mM sodium orthovanadate, 1% Triton X-100) before analysis.

### Noninvasive experiments.

Mice were anesthetized by i.p. injection of 10 mg of xylazine/kg of body weight plus 80 mg/kg ketamine. Mydriatic agents (0.5% tropicamide for 2 min and 10% phenylephrine hydrochloride for 1 min) were applied on the eye for pupil dilation and to block eyebrow movement. The fundi of age-matched CTL and RPE-DKO mice were photographed. OCT was carried out on age-matched CTL and RPE-DKO mice by using an OCT system (Micron III; Phoenix Research Laboratories), according to the manufacturer's recommendations, at 4 months and 1 year.

For scotopic ERGs, age-matched CTL and RPE-DKO mice were dark adapted overnight before anesthesia and the application of the mydriatic agents as described above. For photopic ERGs, mice were exposed to bright light of 3 cd for 10 min. Full-field ERGs were recorded by using the HMsERG system (Ocuscience).

### Immunohistochemistry.

Enucleated eyes were fixed in 4% paraformaldehyde (PFA)–PBS for 45 min, followed by cryoprotection in 30% sucrose–PBS. Ten-micrometer embedded frozen sections were further processed for immunohistochemistry. Briefly, frozen retina sections were blocked in PBS with 3% normal goat serum (Sigma-Aldrich) and 0.2% Triton X-100 (Sigma-Aldrich) for 1 h at room temperature (RT) and incubated with primary antibodies in blocking buffer overnight at 4°C. The following primary antibodies were used: cone arrestin (Merck Millipore), rhodopsin (Rho 1D4, a kind gift of Robert J. Moldy), mOpsin (Chemicon), STRA6 (Abcam), GNAT1 (Santa Cruz Biotechnology), GNAT2 (Santa Cruz Biotechnology), RPE65 (Pin5, a kind gift of Andreas Wenzel), LRAT (Santa Cruz Biotechnology), GFAP (Chemicon), and glycine (ImmunoSolution) antibodies. Sections were incubated again in blocking buffer for 30 min at RT before incubation with the secondary antibodies for 1 h at RT. Secondary or fluorescence-labeled antibodies were a goat anti-mouse antibody–Alexa Fluor 488 conjugate, a goat anti-mouse antibody–Alexa Fluor 594 conjugate, a goat anti-rabbit antibody–Alexa Fluor 488 conjugate, a goat anti-rabbit antibody–Alexa Fluor 594 conjugate, and Oregon Green 488-phalloidin (Thermo Fisher Scientific). Incubation with the secondary antibody alone was used as a negative control. Tissue sections were counterstained with 4′,6-diamidino-2-phenylindole (DAPI) to identify retinal cell layers.

For flat-mount choroid-RPE structures, dissected choroid-RPE sections were mounted onto a coverslip, fixed in 4% PFA–PBS for 45 min, and processed for immunolabeling with Oregon Green 488 as described above. A TUNEL assay (Roche Life Science) was carried out as described previously (23).

### Tissue isolation, mRNA extraction, and protein preparation.

Enucleated eyes from CTL and RPE-DKO mice were dissected under a microscope to exclude extraocular tissues. The cornea, lens, iris, and vitreous body were removed, and the retina was extracted. Either the retina, the RPE, or the RPE-choroid was processed for protein/mRNA isolation.

For mRNA extraction, either the retina or the RPE-choroid was resuspended in TRIzol reagent (ThermoFisher) and stored at −80°C until further handling. RPE mRNA was extracted by using a previously described protocol (73), while retinas and RPE-choroids were extracted by using the following protocol. Briefly, 0.1 volumes of sodium acetate, 1 volume of phenol, and 0.2 volumes of chloroform were added to the tube and mixed, and phase separation was allowed for 15 min on ice. One volume of isopropanol and 2 μl of glycogen (5 μg/μl) were added to the upper aqueous phase and left to precipitate at −20°C overnight. Following centrifugation at 10,000 × *g* for 10 min at 4°C, the precipitate was washed with 75% ethanol (EtOH) and allowed to dry at RT before being resuspended in diethyl pyrocarbonate (DEPC)-treated double-distilled water (ddH_2_O). Quantitative PCR was carried out as described previously (23), using 100 nM primers. The sets of primers used for genotyping (Fig. 1B) and reverse transcription-PCR (RT-PCR) can be found in Table 1.

**TABLE 1:** Primers used for genotyping and qPCR

Purpose	Gene	Primer	Sequence
Genotyping	*Erk2*	B	GCCTTCCAACCTCCTGCTGAACACC
		Wt	GCACCTAACAAAGCTTCACCCAGG
		D	AAGCTTGAGCTCCTCGAGAGATCGGC
		D1	GTACTGGATCCGAGCTCATAACTTCG
		D2	GGGATCAGCTTCAACCTTGCTGGG
	*Erk1*	A	GAAGGAGCCAAGCTGCTATT
		C	AGCAATGACCACATCTGCTA
		K	AACGTGTGGCTACGTACT
	*Cre*	R	CTAATCGCCATCTTCCAGCAGG
		F	AGGTGTAGAGAAGGCACTTAGC
	*rTtA*	R	TCAAACTCGAAGTCGGCCATATCC
		F	CGGCCTTGAATTGATCATATGCGG
qPCR	*Gnat1*	R	ACTGAATGTTGAGGTGGTC
		F	AGAGGATGCTGAGAAGGATG
	*Gnat2*	R	GACTTGAACTCTAGGCACTC
		F	CATCAGTGCTGAGGACAAAG
	*sOpsin*	R	AGGGCCAACTTTGCTAGAAG
		F	TGGTCAACAATCGGAACCAC
	*mOpsin*	R	GGCGCAGCTTCTTGAATCTC
		F	TGAGGATAGCACCCATGCAA
	*Rpe65*	R	AAAGCACAGGTGCCAAATTC
		F	CCCTCCTGCACAAGTTTGAC
	*mRl8*	R	GCTTCACTCGAGTCTTCTTG
		F	ACTGGACAGTTCGTGTACTG
	*Lrat*	R	GACAGCCGAAGCAAGACTGCT
		F	ACGCAGAGCTGAGCAGCAGTT
	*ConeArr*	R	AGTTGTCCAGACCACAGATG
		F	TTGTGCTAGAGGCCAGATTG

Protein isolation from retinas was performed by two successive homogenizations of the retina with syringes (23 gauge and 26 gauge) on ice, followed by 3 freeze-thaw cycles. RPE cell lysates were prepared as previously described (74). Briefly, the incised RPE-choroid was incubated in radioimmunoprecipitation assay (RIPA) buffer (50 mM Tris [pH 8.0], 150 mM NaCl, 1% NP-40, 0.5% sodium deoxycholate, 0.1% SDS) for 10 min with shaking. The choroid was transferred to a different tube, leaving the shredded RPE cells on the tube wall.

For Western blot analysis, extracted retinas were resuspended in lysis buffer (20 mM MOPS [pH 7.0], 2 mM EGTA, 5 mM EDTA, 30 mM sodium fluoride, 60 mM β-glycerophosphate [pH 7.2], 20 mM sodium pyrophosphate, 1 mM sodium orthovanadate, 1% Triton X-100, and 1 M dithiothreitol [DTT] plus protease inhibitors), while the RPE were resuspended in RIPA buffer (50 mM Tris [pH 8.0], 150 mM NaCl, 1% NP-40, 0.5% sodium deoxycholate, 0.1% SDS). All samples were conserved at −80°C until further processing. Extracted cell lysates were separated by SDS-PAGE and transferred onto nitrocellulose membranes (Millipore). The membranes were probed by using the indicated primary antibodies and isotype-matched secondary antibodies conjugated to fluorescent dye (Licor Biosciences) and detected by using the Odyssey imaging system (Licor). The following primary antibodies were utilized: GNAT1 (Santa Cruz Biotechnology), GNAT2 (Santa Cruz Biotechnology), ERK1/2 (Cell Signaling Technology), α-tubulin (Sigma-Aldrich), and RPE65 (Pin5) antibodies. The following secondary bodies were applied: IRDye 680RD (Licor Biosciences) and IRDye 800CW (Licor Biosciences).

### Luciferase and AP-1 binding assays.

Luciferase activity in cell lysates was measured by using a coenzyme A-coupled assay system with luciferin and ATP (Promega). Ten micrograms of cell lysates was mixed with 20 μl of luciferase assay reagent in 4 triplicates. Luminescence was read by using kinetic software on a 384-well-plate spectrophotometer (PerkinElmer). Beta-galactosidase (β-Gal) activity was measured by using a luminescent substrate endpoint assay. Twenty micrograms of lysates was mixed with 50 μl 2× β-Gal buffer (120 mM Na_2_HPO_4_, 80 mM NaH_2_PO_4_, 2 mM MgCl_2_, and 100 mM β-mercaptoethanol [β-ME]) and 50 μl 2× ONPG (2-nitrophenyl-β-d-galactopyranoside). The absorbance at 412 nm was read.

An AP-1 binding assay (TransAM AP-1 kit; Active Motif) was carried out, as described previously (24), on nuclear extracts from ARPE19 cells. Briefly, cells were resuspended in 1 ml cold buffer A (10 mM HEPES [pH 7.9], 10 mM KCl, 1 mM DTT, and protease inhibitors) and placed on ice for 15 min. Next, 12.5 μl of 10% NP-40 was added, and the supernatant was discarded after centrifugation. Nuclei were lysed in buffer B (20 mM HEPES [pH 7.9], 400 mM NaCl, 1 mM DTT, and protease inhibitors) and quantified for protein content. Eight micrograms of the nuclear protein extract or 15 μg of whole-cell protein extracts was used for the AP-1 binding assay according to the manufacturer's instructions.

### AP-1 EMSA.

An AP-1 electrophoretic mobility shift assay (EMSA) was carried out on nuclear extracts from HEK293 cells that were either left untreated or treated with U0126 according to the provider's protocol (Odyssey Infrared EMSA kit; Licor Biosciences). In brief, the nuclear extract was incubated with a solution containing 25 mM DTT–2.5% Tween 20, 1 μg/μl poly(dI·dC), 1% NP-40, 100 mM MgCl_2_, and the IRDye 700 AP-1 consensus oligonucleotide (5′-CGCTTGA*TGACTCA*GCCGGAA-3′) in either the presence or the absence of the AP-1 (in italics) competitor oligonucleotide. Binding was performed for 20 min, after which the sample was loaded onto a 5% native acrylamide gel in the presence of a loading dye. Fluorescence was detected on a Licor Odyssey scanner.

### Retinoid measurement.

Age-matched CTL and RPE-DKO mice were either dark adapted overnight or exposed to bright light of 1,000 mAH for 2 h. For dark conditions, the retina was separated from the RPE-choroid layer. For bright-light conditions, the whole eye was processed for measurement. Ocular tissues were transferred to a 2-ml reaction vial, and 200 μl of 2 M hydroxylamine (pH 6.8) and 200 μl of methanol were added. All steps were conducted under red safety light (>600 nm) to avoid retinoid isomerization. Tissues were mechanically ground by using a Bio-Gen PRO200 homogenizer (Fisher Scientific). The homogenized extracts were allowed to stand for 10 min for oxime formation. Next, 400 μl of acetone and 500 μl of hexane were added. The samples were vortexed, and the aqueous and organic phases were separated by centrifugation at 5,000 × *g* (Minispin Plus; Eppendorf). The organic phase was removed, and extraction was repeated with 500 μl of hexane. Collected organic phases were pooled, dried with a SpeedVac (Eppendorf) at 30°C, and redissolved in HPLC mobile-phase solvent. HPLC analysis was carried out with an Agilent 1260 Infinity Quaternary HPLC system (Santa Clara) equipped with a pump (catalog number G1312C) with an integrated degasser (catalog number G1322A), a thermostated column compartment (catalog number G1316A), an autosampler (catalog number G1329B), a diode array detector (catalog number G1315D), and online analysis software (Chemstation). The analyses were carried out at 25°C by using a normal-phase Zorbax Sil (5-μm, 4.6- by 150-mm) column (Agilent Technologies) protected with a guard column with the same stationary phase. For retinoid separation, the column was developed with 90% hexane and 10% ethyl acetate at an isocratic flow rate of 1.4 ml · min^−1^. For molar quantification of retinoids, the HPLC system was scaled with authentic retinoid standards.

### Electron microscopy.

Enucleated whole mouse eyes were fixed in a 2.5% glutaraldehyde solution (Electron Microscopy Sciences) in phosphate buffer (PB) (0.1 M, pH 7.4) (Sigma-Aldrich) for 90 min at RT. Ocular tissues were then postfixed with a fresh mixture of 1% osmium tetroxide (Electron Microscopy Sciences) with 1.5% potassium ferrocyanide (Sigma-Aldrich) in PB during 1 h 30 min at RT. The samples were then washed three times in distilled water and dehydrated in graded concentrations of an acetone solution (Sigma-Aldrich) (30% for 20 min, 70% for 20 min, 100% for 1 h, and 100% for 2 h). This was followed by infiltration in graded concentrations of Epon (Sigma-Aldrich) (Epon-acetone for 2 h at a 1:3 dilution, 2 h at a 3:1 dilution, and 4 h and 12 h at a 1:1 dilution) and, finally, polymerization for 48 h at 60°C in an oven. Ultrathin sections of 50 nm were cut transversally on a Leica Ultracut microtome (Leica Microsystems) and picked up on a 2- by 1-mm nickel slot grid (Electron Microscopy Sciences) coated with polystyrene film (Sigma-Aldrich). Sections were poststained with 4% uranyl acetate (Sigma-Aldrich) in H_2_O during 10 min, rinsed several times with H_2_O followed by 0.4% Reynolds' lead citrate (75) in H_2_O (Sigma-Aldrich) during 10 min, and rinsed several times with H_2_O.

Micrographs were taken with a Philips (now FEI Company) CM100 transmission electron microscope at an acceleration voltage of 80 kV with a TVIPS TemCam-F416 digital camera (TVIPS GmbH).

### Molecular biology.

pGL2-Basic-RPE65(−450/+39) was a kind gift from Debra Thompson (University of Michigan). pGL3-Basic-LRAT(−268/+257) was a kind gift from Catharine Ross (University of Pennsylvania). pGL2-Basic-RPE65(−450/+39) (33) was digested with BglII and StuI and religated in order to remove the AP-1 site (190 bp segment from the start codon).
